# An insight into the use of telemedicine technology for cancer patients during the Covid-19 pandemic: a scoping review

**DOI:** 10.1186/s12911-024-02507-1

**Published:** 2024-04-19

**Authors:** Esmaeel Toni, Haleh Ayatollahi

**Affiliations:** 1 Student Research Committee, Iran University of Medical Sciences, Tehran, Iran; 2 Health Management and Economics Research Center, Health Management Research Institute, Iran University of Medical Sciences, Tehran, Iran

**Keywords:** Cancer, Covid-19, Scoping review, Telemedicine

## Abstract

**Background:**

The use of telemedicine technology has significantly increased in recent years, particularly during the Covid-19 pandemic. This study aimed to investigate the use of telemedicine technology for cancer patients during the Covid-19 pandemic.

**Methods:**

This was a scoping review conducted in 2023. Various databases including PubMed, Web of Science, Scopus, Cochrane Library, Ovid, IEEE Xplore, ProQuest, Embase, and Google Scholar search engine were searched. All quantitative, qualitative, and mixed-method studies published in English between 2020 and 2022 were included. Finally, the needed data were extracted, and the results were synthesized and reported narratively.

**Results:**

A total of 29 articles were included in this review. The results showed that teleconsultation, televisit, and telerehabilitation were common telemedicine services, and video conferencing and telephone were common technologies used in these studies. In most cases, patients and healthcare providers preferred these services compared to the face-to-face consultations due to their convenience and advantages. Furthermore, the findings revealed that in terms of clinical outcomes, telemedicine could effectively reduce anxiety, pain, sleep disorders, and hospital admission rates.

**Conclusion:**

The findings provided valuable insights into the various telemedicine technologies, services, users’ perspectives, and clinical outcomes in cancer patients during the Covid-19 pandemic. Overall, the positive outcomes and users’ satisfaction showed that the use of telemedicine technology can be expanded, particularly in cancer care. Future research needs to investigate both clinical and non-clinical effectiveness of using various telemedicine services and technologies for improving cancer care delivery, which can help to develop more successful strategies for implementing this technology.

## Background

Cancer is a widespread public health concern, with more than 19 million new cancer cases and about 10 million cancer-related deaths reported worldwide in 2020 [1, 2]. The International Agency for Research on Cancer (IARC) reported that globally, one out of five people is susceptible to develop cancer during their lifetime, and one out of every eight men and one out of every 11 women may die from cancer. It seems that an increase in the global population, diseases, and economic factors are the main risk factors for increasing the prevalence of cancer [3].

Recent studies have shown that cancer patients face an increased possibility of being diagnosed with Covid-19 and they experience more severe clinical manifestations [4]. Moreover, the mortality of cancer patients due to Covid-19 is higher compared to the people without cancer [5]. Cancer patients are more affected by the side effects of Covid-19, and the lack of adequate and timely care for these patients may lead to an increase in their mortality rate [4].

There are a number of risk factors that cause cancer patients being affected by Corona virus transmission. These risk factors include the progression of the disease, the suppressive nature of the immune system, frequent visits to hospitals for active chemotherapy, radiotherapy, immunotherapy, targeted therapy and immunosuppressant for bone marrow transplantation [6, 7]. As a result, telemedicine tools have been recommended to reduce disease transmission rates while continuing medical care provision remotely, particularly during the pandemic [8, 9].

Although telemedicine technology is not a novel method for providing cancer patients with a variety of healthcare services, such as diagnosis, consultation, treatment, and home care [10], the use of this technology has increased during the Covid-19 pandemic, mainly due to the high workload on the healthcare system and the provision of global strategies such as social distancing and quarantine requirements. These reasons encouraged cancer patients to use telemedicine technologies instead of receiving an in-person visit in medical centers [11, 12]. The use of telemedicine has a number of advantages. For example, it can help to reduce the waiting times compared to the traditional in-person visits at healthcare centers [13]. However, despite the advantages and positive users’ attitudes, there are still concerns over the lack of face-to-face communication and the inability to conduct physical examinations [14].

So far, several review studies have been conducted; however, a few of them discussed the use of telemedicine technology by cancer patients during the Covid-19 pandemic [15–17]. The review studies in this area were also scarce and their objectives were different [15, 17, 18]. Therefore, in the current study, we aimed to conduct a scoping review to systematically investigate and consolidate the evidence related to the application of telemedicine technology for cancer patients during the Covid-19 pandemic. By adopting a scoping review methodology, we could provide a comprehensive understanding of different types of technology, services, users’ perspectives, and clinical outcomes of using telemedicine technology within the context of cancer care during the pandemic. The results can be useful for future research, further technology development, and response to the unique needs of cancer patients especially during the pandemic.

## Methods

This scoping review was conducted in 2023 and different types of research, in which telemedicine intervention was used for cancer patients during the Covid-19 pandemic and its outcome was evaluated, were reviewed. Unlike systematic reviews, which typically focus on a narrow research question and employ a rigorous method to assess the risk of bias and quality, scoping reviews provide a broad overview of the available literature without a formal assessment of these factors [19]. To achieve this goal, Arksey and O’Malley’s framework [19] was used. Before conducting this research, ethics approval (IR.IUMS.REC.1401.392) was obtained from the National Ethics Committee of Biomedical Research.

### Stage 1: identifying the research question

Before developing a search strategy for review studies, the research question should be explained clearly. The initial literature review suggested that the literature about the use of telemedicine technology for cancer patients during the Covid-19 pandemic was scarce; therefore, we generated research questions as follows:

- How was telemedicine technology used for cancer patients during the Covid-19 pandemic?
- What was the outcome of telemedicine technology used for cancer patients compared to in-person visits?

### Stage 2: identifying relevant studies

To identify relevant studies, eight databases including PubMed, Web of Science, Scopus, Cochrane Library, Ovid, IEEE Xplorer, ProQuest, and Embase as well as the Google Scholar search engine were searched. The search timeframe was between 1st January 2020 and 31th December 2022 to access all relevant studies published during the pandemic. The search strategy consisted of three main terms: “Covid-19”, “Telemedicine” and “Cancer,” along with their corresponding synonyms and MeSH terms. All of them were combined using AND OR logical operators to create search strategies for different databases (Table 1). The reference list of the selected studies and their citations were also searched to ensure that all relevant studies were included.

**Table 1:** Search strategies

Database	Search strategy	Number of records
Web Of Science	(TI=(( ( ( “Telemedicine” OR “mhealth” OR “Telehealth” OR “telerehabilitation” ) AND ( “cancer” OR “tumor” OR “neoplasm” OR “medical oncology” ) AND ( “Covid-19” OR “coronavirus” OR “SARS-COV-2” OR “Sever acute respiratory syndrome coronavirus 2” ) ) ) )) OR AB=(( ( ( “Telemedicine” OR “mhealth” OR “Telehealth” OR “telerehabilitation” ) AND ( “cancer” OR “tumor” OR “neoplasm” OR “medical oncology” ) AND ( “Covid-19” OR “coronavirus” OR “SARS-COV-2” OR “Sever acute respiratory syndrome coronavirus 2” ) ) ) ) and 2020 or 2021 or 2022 (Publication Years) and English (Languages)	563
PubMed	((“Telemedicine“[MeSH Terms] OR “mhealth“[Title/Abstract] OR “Telehealth“[Title/Abstract] OR “telerehabilitation“[MeSH Terms]) AND (“cancer“[Title/Abstract] OR “medical oncology“[MeSH Terms] OR “tumor“[Title/Abstract]) AND (“Covid-19“[MeSH Terms] OR “coronavirus“[MeSH Terms] OR “SARS-COV-2“[MeSH Terms] OR “Sever acute respiratory syndrome coronavirus 2“[Title/Abstract])) AND ((2020:2022[pdat]) AND (english[Filter]))	569
Scopus	TITLE-ABS( ( ( “Telemedicine” OR “mhealth” OR “Telehealth” OR “telerehabilitation” ) AND ( “cancer” OR “tumor” OR “neoplasm” OR “medical oncology” ) AND ( “Covid-19” OR “coronavirus” OR “SARS-COV-2” OR “Sever acute respiratory syndrome coronavirus 2” ) ) ) AND PUBYEAR > 2019 AND PUBYEAR < 2023 AND ( LIMIT-TO ( LANGUAGE,“English” ) )	565
Embase	(‘telemedicine’:ab, ti OR ‘mhealth’:ab, ti OR ‘telehealth’:ab, ti OR ‘telerehabilitation’:ab, ti) AND (‘cancer’:ab, ti OR ‘tumor’:ab, ti OR ‘neoplasm’:ab, ti OR ‘medical oncology’:ab, ti) AND (‘Covid-19’:ab, ti OR ‘coronavirus’:ab, ti OR ‘sars-cov-2’:ab, ti OR ‘sever acute respiratory syndrome coronavirus 2’:ab, ti) AND [english]/lim AND [2020–2022]/py	974
Ovid Medline	((“Telemedicine” or “mhealth” or “Telehealth” or “telerehabilitation”) and (“cancer” or “tumor” or “neoplasm” or “medical oncology”) and (“Covid-19” or “coronavirus” or “SARS-COV-2” or “Sever acute respiratory syndrome coronavirus 2”)).ti. or ((“Telemedicine” or “mhealth” or “Telehealth” or “telerehabilitation”) and (“cancer” or “tumor” or “neoplasm” or “medical oncology”) and (“Covid-19” or “coronavirus” or “SARS-COV-2” or “Sever acute respiratory syndrome coronavirus 2”)).ab. Language: English Publication Date: 2020-01-01 to 2022-12-30	522
IEEE Xplore	(“Telemedicine” OR “mhealth” OR “Telehealth” OR “telerehabilitation”) AND (“cancer” OR “tumor” OR “neoplasm” OR “medical oncology”) AND (“Covid-19” OR “coronavirus” OR “SARS-COV-2” OR “Sever acute respiratory syndrome coronavirus 2”)	4
Cochrane library	42 Trials matching (“Telemedicine” OR “mhealth” OR “Telehealth” OR “telerehabilitation”) AND (“cancer” OR “tumor” OR “neoplasm” OR “medical oncology”) AND (“Covid-19” OR “coronavirus” OR “SARS-COV-2” OR “Sever acute respiratory syndrome coronavirus 2”) in Record Title OR (“Telemedicine” OR “mhealth” OR “Telehealth” OR “telerehabilitation”) AND (“cancer” OR “tumor” OR “neoplasm” OR “medical oncology”) AND (“Covid-19” OR “coronavirus” OR “SARS-COV-2” OR “Sever acute respiratory syndrome coronavirus 2”) in Abstract - (Word variations have been searched)	42
ProQuest	abstract(((“Telemedicine” OR “mhealth” OR “Telehealth” OR “telerehabilitation”) AND (“cancer” OR “tumor” OR “neoplasm” OR “medical oncology”) AND (“Covid-19” OR “coronavirus” OR “SARS-COV-2” OR “Sever acute respiratory syndrome coronavirus 2”))) OR title(((“Telemedicine” OR “mhealth” OR “Telehealth” OR “telerehabilitation”) AND (“cancer” OR “tumor” OR “neoplasm” OR “medical oncology”) AND (“Covid-19” OR “coronavirus” OR “SARS-COV-2” OR “Sever acute respiratory syndrome coronavirus 2”)))Limits applied Databases: • Coronavirus Research Database • Publicly Available Content Database *These databases are searched for part of your query.* Narrowed by: Entered date: 2020–2022; Language: English	149
Google Scholar	allintitle: (“cancer” OR “tumor” OR “oncology”) AND (“Covid-19” OR “SARS-COV-2” OR “coronavirus”) AND (“Telemedicine” OR “Telehealth” OR “Telecare” OR “telerehabilitation”) (2020–2022)	113

### Stage 3: study selection

The current study utilized specific criteria for including and excluding articles during the selection process. The inclusion criteria consisted of original articles published in English in which quantitative, qualitative, or mixed methods approaches were used to evaluate telemedicine interventions for cancer patients during the Covid-19 pandemic, and their results were compared with the in-person visits.

The exclusion criteria encompassed review articles, letters to the editor, protocols, and studies that did not involve the evaluation of telemedicine technology by cancer patients or their care providers. Furthermore, studies that were not published in the English language, those that focused on cancer survivors instead of individuals currently undergoing treatment, those that did not report clinical outcomes or did not gather users’ perspectives (patients or care providers), abstract-only documents, and full-texts that were not accessible were all excluded from this review study.

### Stage 4: charting the data

To perform a complete search on the subject, all related keywords and MeSH terms were identified and combined. The retrieved studies were organized using EndNote software, and after removing duplicates, the remaining articles were screened in terms of the title and abstract relevancy to the aim of the study. The full text of eligible studies was then retrieved and reviewed. Both authors contributed to screen the articles independently and any disagreements between them were resolved via discussion.

The required data were extracted using a data extraction form and included the author(s) name, country of the study, year of publication, study objective, type of telemedicine technology, type of cancer, type of services (such as diagnosis, treatment, consultation, screening or home care), users’ (patients’ and healthcare providers’) perspectives regarding the use of telemedicine technology for cancer patients during the Covid-19 pandemic, and clinical outcomes.

### Stage 5: collating, summarizing, and reporting the results

The extracted data were tabulated, summarized, and reported narratively. Results were reported by categorizing studies according to the type of services, type of technology, patients’ and providers’ opinions, and clinical outcomes.

## Results

**Fig. 1:**
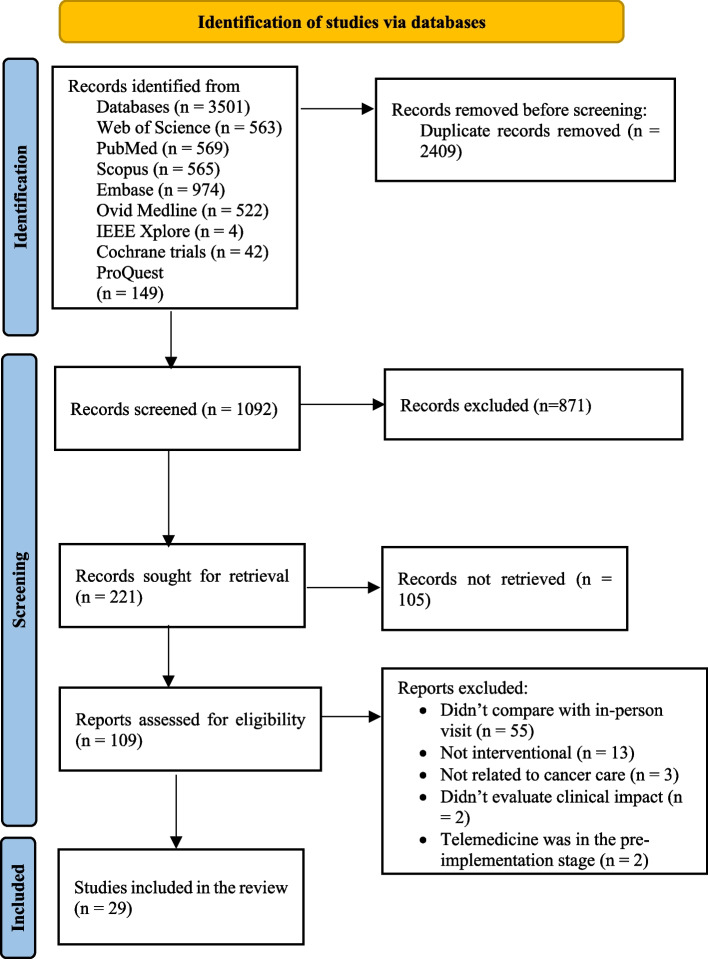
Process of selecting articles

Initially, 3501 articles were retrieved and after completing the screening process based on the PRISMA-ScR statement and applying the inclusion and exclusion criteria, 29 studies [20–48] were identified for the final review. Figure 1 shows the process of selecting articles.

## Characteristics of the selected studies

The results showed that most studies (*n* = 19) were conducted in the United States [20, 22–25, 28, 30–36, 39–41, 43, 44, 47]. Other studies were completed in the United Kingdom (*n* = 3) [27, 37, 42], Italy (*n* = 2) [26, 48], Australia (*n* = 2) [29, 45]. Saudi Arabia (46), China (21), and Canada (38) each contributed to publish one relevant study. Most of the article (*n* = 17) were published in 2022 [32–48]. Table 2 shows a summary of the articles included in the study.

**Table 2:** Summary of the selected studies

No	Author(s)	Country/year	Objective	Disease	Type of telemedicine services	Telemedicine modality	Users’ opinions	Clinical impact
1	Darcourt et al. [20]	USA/2021	To evaluate the use of telemedicine amid the Covid-19 pandemic in patients with cancer and assess barriers to its implementation	All types of cancer	Teleconsultation	Video conference (Using MyChart video platform)	• Most patients were satisfied (92.6%), and 62.5% of physicians were satisfied from teleconsultation. • Patients were more eager to use of telemedicine compared to in-person follow up visits (*P* < .0001) • Limiting physical examinations, physician-patient interaction, medical liability, quality of care, and getting significant data are physicians' concerns about the use of video visits.	Not reported
2	Chen et al. [21]	China/2021	To investigate the effectiveness of telerehabilitation on the short-term quality of life of patients after esophageal cancer surgery during Covid-19.	Esophageal cancer	Telerehabilitation	Video conference, messaging (Using WeChat platform), and remote monitoring	Not reported	• Use of telerehabilitation significantly relieved patients pain (*P* < .001) • Telerehabilitation visits had much lower scores in sleep disturbance, appetite loss, financial impact, swallowing saliva, choking, and cough compare to in-person visits.
3	Shaverdian et al. [22]	USA/2021	To assess cancer patient experience with telemedicine in routine radiation oncology practice to determine satisfaction, quality of care, and opportunities for optimization.	All types of cancer	Teleconsultation	Video conference, or telephone	• Patients overall preferred teleconsultation compared to in-person visits. • Teleconsultation deceases treatment-related costs compared to in-person visits.	Not reported
4	Narayanan et al. [23]	USA/2021	To report the feasibility of conducting integrative oncology physician consultations via telehealth in 2020 and compare patient characteristics to prior in-person consultations undertaken in 2019.	All types of cancer	Teleconsultation	Video conference, or telephone (Using Zoom platform)	Not reported	• Teleconsualtion use significantly lower anxiety, appetite, depression, drowsiness, fatigue, financial distress, and pain scores compare to in-person visits.
5	Granberg et al. [24]	USA/2021	To identify the factors influencing patient acceptability of video visits for medical oncology care before and at the onset of the expansion of telehealth because of the Covid-19 pandemic.	All types of cancer	Televisit	Video conference	• Televisit improved patient convenience and experience by eliminating travel, reducing risk of Covid-19 exposure, and increasing the length of visits. • Limiting physical examinations, privacy concerns, e-health illiteracy, and reducing patient-provider communications are televisit use barriers.	• Televisit reduce anxiety
6	Heyer et al. [25]	USA/2021	To identify medical oncology health professionals’ perceptions of the barriers to and benefits of telehealth video visits.	All types of cancer	Televisit	Video conference (Using JeffConnect platform)	• Televisit is limiting patients' physical examination, patient-provider connection, and sensitive conversations compare to in-person visits • Televisit is facilitate more patient follow-up, availability of lab results, lowering communicable disease, transportation, and increase patient responsibilities.	Not reported
7	Picardo et al. [26]	Italy/2021	To analyze oncological patients’ perception of telemedicine in the Covid-19 pandemic during follow-up visits for gynecological and breast cancers.	Gynecological and breast cancers	Televisit	Telephone	• Young women with pelvic cancer had a better perception of telemedicine compared to older ones. • Low-educated women with breast cancer had a more positive perception of telemedicine enhanced their care.	Not reported
8	Zhu et al. [27]	UK/2021	To assess patient satisfaction with the head and neck cancer telephone triage service during the Covid-19 pandemic.	Head and neck cancer	Televisit	Telephone	• Consulting through telemedicine is more effective than in-person visits (*P* < .001). • Healthcare services is more accessible through telemedicine than in-person visits (*P* = .01). • Using telephone delays diagnosis and hinders communication. • Telemedicine is limiting doctor-patient relationship. • Telephone consultation is safe, easy, accessible and cost saver during Covid-19 pandemic.	• Telephone triage may induce more fear and anxiety.
9	Kotsen et al. [28]	USA/2021	To examine the effect of rapid scaling of tobacco treatment telehealth on cancer patient engagement, as measured by attendance rates for in-person counseling visits versus remote telehealth counseling visits during the Covid-19 outbreak.	All types of cancer	Televisit	Telephone	• The use of telemedicine is more significant to appointment completion than in-person appointments (*P* < 0.001). • Telemedicine appointments completion during the Covid-19 pandemic had increased by 2.3 times compared to in-person visits (*P* < 0.001).	Not reported
10	Watson et al. [29]	Australia/2021	To assess the efficacy and safety of telephone clinics in delivering care to established oncology patients and assess patient and health professionals’ preference (telephone vs. face-to-face clinics) during the Covid-19 pandemic.	All types of cancer	Televisit	Telephone	• Telemedicine saves patients’ money and time, increases visit time, and Telemedicine is limiting patients' physical and vital sign examinations, and reduce patient-provider connection compare to in-person visits	• Before the introduction of telemedicine, patients' mortality post-systemic therapy was significantly high (*P* = .008). • Telemedicine reduce patients’ anxiety by make easier to receive bad news compared to in-person visits.
11	Aghedo et al. [30]	USA/2021	To transition in-person multidisciplinary team to a telehealth format and to assess early outcomes for colorectal cancer patient and physician satisfaction during the Covid-19 pandemic.	Colorectal cancer	Teleconsultation	Video conference (Using Zoom platform	• Physicians were satisfied with teamwork, communication, and quality of care. • Patients reflected a high degree of satisfaction with the easy-to-use of telemedicine, audio-video quality, and quality of communication.	Not reported
12	Fassas et al. [31]	USA/2021	To examine current preferences and barriers for telemedicine among patients with head and neck cancer in the Covid-19 era.	Head and neck cancer	Teleconsultation	Video conference (Using Zoom platform)	• Patients were more comfortable in use of teleconsultation (*P* = .028) and with an assistance (*P* = .007) compared to in-person visits.	Not reported
13	Uppal et al. [32]	USA/2022	To measure short-term outcomes of cancer patients with postoperative telemedicine visits compared with in-person visits	All types of cancer	Teleconsultation	Video conference	• Use of telemedicine takes fewer additional visits compared to in-person visits (P = .01)	• Use of telemedicine takes shorter surgical length of stay (P = .001)
14	Mackwood et al. [33]	USA/2022	To study factors that influenced telemedicine uptake and sustained use in outpatient oncology clinics at a USA cancer center to inform future telemedicine practices	All types of cancer	Teleconsultation	Video conference, and telephone (Using Zoom platform)	• Reimbursement, licensing regulations, and access to local patient's medical records are providers concern. • Clinical workflow compatibility, internet connectivity, and patients' technical illiteracy are teleconsultation barriers. • Teleconsultation had overcome in-person geographical, time, and workload barriers. • The Covid-19 pandemic had a significant influence on the use of teleconsultation.	Not reported
15	Alpert et al. [34]	USA/2022	To describe oncology clinicians’ experiences with teleoncology and to uncover its benefits and challenges during the first 10 months of the Covid-19 pandemic.	All types of cancer	Teleconsultation	Video conference (Using Zoom platform)	• Telemedicine reduces in-person visits, travel, financial burden, and risk of Covid-19 exposure. • Telemedicine facilitates family member participation and makes patients and their environments visible. • The internet connection, unfamiliarity with telemedicine, conducting physical exams, and meeting expectations about appointment times are provider’s challenges.	Not reported
16	Hadley et al. [35]	USA/2022	To identify medical oncology providers’ perceptions of telehealth video visits as influenced by the Covid-19 pandemic.	All types of cancer	Televisit	Video conference (Using JeffConnect platform)	• Televisits increased patients' and providers' comfort and their willingness to engage during the Covid-19 pandemic compared to in-person visits. • Access to unreliable technology and the internet, reticence to change, inability to maintain a robust provider-patient relationship, lack of physical examination, and fear of faulty diagnoses or inappropriate treatment are televisit barriers.	Not reported
17	Waseem et al. [36]	USA/2022	To investigate factors associated with successfully accessing and completing telemedicine visits and the association between telemedicine visit success and clinical outcomes among patients with thoracic cancer during the Covid-19 pandemic.	Thoracic cancer	Televisit	Video conference, or telephone	Not reported	• Televisits reduced odds of urgent care visits, and hospitalization compared to in-person visits.
18	Brady et al. [37]	UK/2022	To evaluate and co-design rehabilitation services via telemedicine services to meet the complex needs of our patients and careers at a tertiary cancer center.	Head and neck, Gastrointestinal, breast, and hematology cancer	Telerehabilitation	Video conference, or telephone	• Telerehabilitation saves both patients and hospital travel costs and time and makes visits more flexible and patient-centered compared to in-person visits. • Both patients and careers had privacy and security concerns, inappropriate access to use, communication difficulties, and lack of training.	Not reported
19	Khan et al. [38]	Canada/2022	To understand patient experiences from their perspective regarding telehealth interaction for swallowing therapy during radiation therapy.	Head and neck cancer	Telerehabilitation	Video conference, telephone, or both (Using MS Teams or WebEx platforms)	• Telerehabilitation was identified as easy to use due to participants' good internet connection, minimal required preparation, and family member support. • Saving time and money due to travel elimination, reducing in-person session anxiety, and protection against the Covid-19 pandemic were the most expressed benefits of telerehabilitation. • Lack of previous telerehabilitation experience, access to optimal equipment, and physical examination were the most limiting use factors.	Not reported
20	Breen et al. [39]	USA/2022	To examine patient experiences with and preferences for telehealth at a cancer genetic counseling clinic throughout the first six months of the Covid-19 pandemic.	All types of cancer	Teleconsultation	Video conference, telephone, or both	• Most of the patients felt grateful when received the scheduled teleconsultation appointment notification. • Some patients expressed technical and low-quality of care concerns before teleconsultation appointments. • Most patients are satisfied with teleconsultation appointments, ease of use, and the quality of audio/visual during the appointment.	Not reported
21	Mackwood et al. [40]	USA/2022	To characterize the use of telemedicine for oncology care over the course of the Covid-19 pandemic in Northern New England with a focus on factors affecting trends.	All types of cancer	Televisit	Video conference, or telephone	• Televisit decreases emergency room and hospital admission rates compared to in-person visits. (*P* < .001)	Not reported
22	Turner et al. [41]	USA/2022	To explore oncology healthcare providers' and professionals’ experiences with telehealth implementation during the Covid-19 pandemic.	All types of cancer	Teleconsultation	Video conference, or telephone (Using Zoom platform)	• Teleconsultation increased patient receptivity to information, willingness to initiate discussion, coordination with caregivers and external/internal healthcare providers. • Lack of physical examinations, data (e.g. patient-reported outcomes), electronic health record integration, information technology support, patient education, and workflow optimization are the most expressed teleconsultation challenges.	Not reported
23	Grant et al. [42]	UK/2022	To elucidate the perceptions and opinions of cancer patients at St. Bartholomew’s Hospital and The Royal Free Hospital in regard to this recent and rapid transition to teleclinics.	All types of cancer	Televisit	Video conference, or telephone	• Telemedicine saved patients time and reduced fatigue from travel • Using telephone-based televisits may make the struggle to patients with hearing/language difficulties.	• Using telemedicine to confirm systemic anti-cancer therapy is acceptable to patients
24	Ackroyd et al. [43]	USA/2022	To describe the use of telemedicine in gynecologic oncology and identify patient characteristics associated with telemedicine use during Covid-19.	Gynecologic cancers	Televisit	Video conference, or telephone	• Patients who had at least one televisit were more likely to have multiple visits than only attending in-person visits. (*P* < 0.01)	Not reported
25	Mojdehbakhsh et al. [44]	USA/2022	To evaluate gynecologic cancer patients’ satisfaction with telemedicine visits over a one-year period during the Covid-19 pandemic.	Gynecologic cancers	Televisit	Video conference, or telephone	• Most of patients were satisfied from quality of technology, personal comfort, length-of-visit, treatment explanation, and overall experience. • Televisit is limiting patient-providers interaction, and physical examinations.	Not reported
26	Collins et al. [45]	Australia/2022	To evaluate perceptions of telehealth through a dyadic exploration of matched cancer patient-and clinician-reported acceptability data and to explore factors that may predict greater suitability for telehealth.	All types of cancer	Teleconsultation	Video conference, or telephone	• Most patients and clinicians were satisfied with the use of teleconsultation. • Based on clinicians' views, the use of teleconsultation for young patients, with higher performance status, or low-stage of cancer was more acceptable.	Not reported
27	Almouaalamy et al. [46]	Saudi Arabia/2022	To investigate the effect of teleclinics on palliative care patients during the Covid-19 pandemic.	All types of cancer	Televisit	Video conference	Not reported	• Patients with full code status were relatively less likely to be admitted (*P* < .001) or go to the emergency room (*P* = .022)
28	Tang et al. [47]	USA/2022	To evaluate surgical telehealth utilization and outcomes for newly diagnosed breast cancer patients during the Covid-19 pandemic.	Breast cancer	Teleconsultation	Video conference, and telephone	• Patients with a teleconsultation had a higher number of subsequent office visits compared to an initial office visit. (P < .001)	• Teleconsultation takes a shorter time from biopsy to first surgical consultation compared to in-person visits. (*P*= .01)
29	Pardolesi et al. [48]	Italy/2022	To report the results of the initial experience of the SmartDoc Project, a telemedicine program activated in a cancer center at the epicenter of the Covid-19 pandemic onset in Italy.	Lung cancer	Teleconsultation	Video conference (Using MS Teams platform)	• Most patients were highly satisfied with teleconsultation compared to in-person visits. • Most patients choose telemedicine over traditional in-person consultation due to fear of Covid-19 virus transmission.	Not reported

## Types of telemedicine technology

Video conferencing [20–25, 30, 32–48], telephone [22, 23, 26–29, 33, 36–45, 47] and messenger applications such as WeChat [21] were the most commonly used technologies. The platforms used for video conferencing were Zoom [23, 30, 31, 33, 34, 41], Jeff Connect [25, 35], Microsoft Teams [38, 48], WeChat [21], and MyChart [20]. Additionally, the results showed that using video conferencing helped with discussing various treatment plans [20, 22, 23, 31, 32, 34, 39, 41, 45, 46, 48] and patients’ symptoms [20, 21, 23, 24, 36, 37, 42, 46, 47], as well as receiving advice and self-care recommendations from the physicians [20–23, 25, 30, 33, 35, 36, 43, 45, 47, 48].

Similarly, telephone-based telemedicine services helped with receiving advice about the clinical symptoms of cancer patients [23, 29, 36, 42, 47], scheduling the next appointments [27, 28, 39], and receiving regular consultation services [22, 23, 26–29, 33, 36–45, 47]. In addition, for cancer patients who were in the initial stages of the disease, telephone consultation was an effective way to receive continuous care and support [45]. In Chen et al.‘s study, the results showed that the messenger applications facilitated message exchange for the rehabilitation of cancer patients during the Covid-19 pandemic [21].

### Types of telemedicine services

Teleconsultation (*n* = 13) [20, 22, 23, 30–34, 39, 41, 45, 47, 48], televisit (*n* = 13) [24–29, 35, 36, 40, 42–44, 46], telerehabilitation (*n* = 3) [21, 37, 38] and telemonitoring (*n* = 1) [21] were the main types of telemedicine services provided to the cancer patients. Teleconsultation and televisit were the most common types of services that allowed cancer patients to communicate with healthcare providers without having to leave their homes and helped to eliminate the need to visit health centers and minimize the risk of virus exposure [20, 22, 23, 30–34, 39, 41, 45, 47, 48]. Televisit was used to provide ongoing care through symptom management and drug prescription for cancer patients [24–29, 35, 36, 40, 42–44, 46].

Telerehabilitation was another telemedicine service which was used to provide physiotherapy [21, 38] and occupational therapy [37] for cancer patients during the Covid-19 pandemic Telemonitoring was used to continuously track vital signs and provided feedback regarding the progress of patient rehabilitation, which could be useful in motivating and improving the effectiveness of telerehabilitation in the era of Covid-19 [21].

### Patients’ and healthcare providers’ perspectives

According to the results, in some studies, patients were highly satisfied with telemedicine services [20, 30, 39, 44, 45, 48]. Patient satisfaction was specifically related to the audio/video quality [30, 39], ease of communication [30, 38], technology ease of use [30, 38, 39], comfort, appropriate length of visit, and overall experience of using telemedicine technology compared to the in-person visits [44]. In some studies, physicians were also highly satisfied with the use of telemedicine technology in the field of cancer care [20, 30, 45]. For example, Aghdo et al. showed that physicians were satisfied with the teamwork, increased communication, and quality of care which were resulted from using telemedicine technology [30].

The findings also showed that most patients were willing to use telemedicine technology compared to the in-person consultations [20, 22, 25, 27, 28, 31, 32, 35, 39, 47]. Their main reasons included reducing the number of in-person visits [32, 43], feeling more comfortable [31, 35], improving the follow-up services [25, 35], and increasing the number of successful appointments [28, 47] compared to the in-person visits.

The main benefits of telemedicine for patients included eliminating unnecessary travels [24, 25, 33, 34, 37, 42], saving time [29, 33, 37, 38, 42] and cost [21–23, 27, 29, 34, 37, 38], better and easier access to medical services [24, 25, 27], facilitating family members engagement [34], more flexibility in making appointments [29, 37], increasing the duration of visits [24, 29], improving adherence to medical advice [41] and reducing the risk of exposing to Covid-19 [24, 25, 27, 33, 34, 48]. Healthcare providers could also benefit from reducing the workload [33] and collaborating with other caregivers and specialists inside and outside the hospital [41].

There were also some challenges to the use of telemedicine technology by patients and their care providers [20, 24, 25, 27, 29, 33–35, 37–39, 41, 42, 44]. For instance, inability to conduct physical examinations and monitor the clinical symptoms of patients [20, 24, 25, 29, 34, 35, 38, 41, 44], limited physician-patient interactions [20, 24, 25, 27, 29, 35, 44], concerns over data privacy and security [24, 25, 35, 37], and issues with Internet connectivity and IT support [33, 34, 37, 39, 41] were some of these challenges.

Patients were concerned about the lack of access to communication tools and appropriate equipment (such as the Internet and personal computers) [38], low technical skills and e-literacy [24, 33, 34, 37, 38, 41], medical responsibility [20], and care quality through telemedicine [20, 37, 39]. Similarly, healthcare providers were concerned with the compatibility of technology with their common workflows [33, 35, 41], limited access to patient information and medical records [20, 33, 41], and reimbursement difficulties [20, 33, 41] as challenges hindering them from using telemedicine effectively. The results of some studies also showed that telephone consultations may cause several challenges, such as delay in diagnosis [27] and limiting physician-patient interactions [27]. In addition, it can be challenging for patients with language or hearing impairments to use this technology effectively [42].

### Clinical outcomes

In addition to the non-clinical benefits of telemedicine technology, several studies have demonstrated that patients who received care through this technology experienced positive clinical outcomes, such as reduction in anxiety [24, 27, 29, 38], pain [21, 23], sleep disorders [21, 23], fatigue [23], and uncontrolled appetite [21, 23] compared to those who had in-person visits. For instance, Chen et al. found that telerehabilitation was associated with reduced difficulty swallowing saliva, asphyxia, and cough among patients with head and neck cancers [21]. Other positive clinical outcomes of telemedicine over in-person consultations included reducing hospitalization and emergency care visits [33, 36, 46], reducing mortality rates [29], and improving treatment outcomes [42] for patients undergoing systemic anti-cancer treatments. Additionally, telemedicine can lead to faster biopsy consultation and diagnosis processes compared to the in-person visits [47].

### Synthesis

Overall, the synthesis of the results showed that telemedicine was widely used for cancer patents during the Covid-19 pandemic. Like other chronic diseases, teleconsultation and televisit were the most common methods for delivering healthcare services. Although patients and healthcare providers were generally satisfied with this technology, a number of technical and non-technical challenges are still remaining which need further attention.

## Discussion

During the Covid-19 pandemic, telemedicine was identified as a valuable intervention to facilitate the provision of care services for cancer patients [17]. This study aimed to investigate the use of telemedicine technology for cancer patients during the Covid-19 pandemic. The results revealed that video conferencing, telephone consultation, messaging applications, such as WeChat, and telemonitoring were commonly used technologies. Popular platforms included Zoom, Jeff Connect, Microsoft Teams, WeChat, and My Chart. Teleconsultation, televisit, and telerehabilitation were among the most frequently offered services. Overall, both healthcare providers and patients were highly satisfied with the telemedicine technology. The benefits of telemedicine included eliminating unnecessary travel time and costs, facilitating access to medical services, and reducing the risk of exposure to Covid-19. However, potential challenges such as limited access to, or proficiency with the communication tools or equipment, lack of technical skills, low level of e-literacy, and concerns over quality assurance are still remaining. Additionally, the findings suggested that using telemedicine helped to reduce anxiety levels, pain, sleep disorders, fatigue, and uncontrolled appetite while decreasing emergency care needs in many cases.

Several studies have demonstrated that video conferencing is an effective method for delivering virtual face-to-face consultations to the cancer patients during the Covid-19 pandemic [17, 49]. This technology has been used to facilitate screening, counseling, rehabilitation, mental health support [16], while improving clinical outcomes [50]. Additionally, video conferencing can help to reduce travel time and costs. However, some potential challenges, such as poor Internet connectivity, concerns over privacy and security, and limitations in conducting physical examinations or remote diagnostic tests still need further attentions [51]. Fortunately, the use of popular platforms such as WhatsApp, Zoom, and Skype has helped with conducting video conferencing sessions between healthcare providers and patients [52].

Several studies have indicated that telephone counseling can be an effective tool in providing palliative care for cancer patients [53] and can assist with providing mental health support to manage their anxiety and stress associated with the treatments [54]. Furthermore, with the increasing use of smartphones, messaging applications like WhatsApp, Viber, and WeChat [55] have been used to provide palliative support and rehabilitation services to cancer patients, leading to better management of clinical symptoms [56]. In addition, the use of these platforms has increased awareness among cancer patients regarding the treatment options during Covid-19 [56], and improved their functional capacity, psychological well-being, and overall quality of life [57].

Our study findings are consistent with other studies in which teleconsultation has been identified as one of the most commonly used telemedicine services for cancer patients during the Covid-19 pandemic [17]. Teleconsultation has proven to be an efficient and effective tool for receiving medical care, and timely palliative and emotional counseling outside of the hospital settings [58]. Similarly, televisit services have been used to maintain ongoing care delivery and support cancer patients during the pandemic. These virtual visits offered a valuable alternative for urgent medical care needs when in-person consultations were not feasible due to the social restrictions [59, 60] In addition, telerehabilitation services have demonstrated benefits in improving muscle strength, functional ability, pain relief, and reducing sleep disorders among cancer patients undergoing surgery or chemotherapy during the pandemic [61, 62]. Telemonitoring was also found to be effective in tracking the vital signs of cancer patients and providing feedback on rehabilitation progress during the pandemic [21, 63]. This finding is supported by Steimer et al.‘s study which showed that telemonitoring improved clinical outcomes and vital signs, and reduced hospitalization rates compared to the in-person visits among cancer patients [64].

Our research findings demonstrated that telemedicine services have also increased patients’ and healthcare providers’ satisfaction mainly due to maintaining quality of care and positive clinical outcomes compared to the in-person visits [60, 65, 66]. This positive feedback can help to accelerate development and implementation of telemedicine technology for cancer patients [67]. Although the effectiveness of telemedicine may vary depending on various factors, such as patient characteristics, type of cancer and its stage [14, 68], patients still use this type of services for various reasons, including reduced waiting times/costs [69], ease of accessibility/use, reduction in physician visits, improved treatment follow-up, and increased comfort levels [70]. Apart from their positive feedback, their concerns need to be addressed, too. It is crucially important to address infrastructure deficiencies and resolve technical issues, and reimbursement challenges for encouraging continuous use of telemedicine services [71, 72].

### Implications for practice

This research provided an insight into the use telemedicine for cancer care during the Covid-19 pandemic. According to the findings, it is essential to customize telemedicine platforms to address users’ concerns regarding the quality of audio and video materials, as well as the ease of communication. Adapting services to meet the specific needs of patients at various stages of cancer care improves their overall satisfaction. Moreover, it is crucial to address technical obstacles, such as the Internet connectivity issues, in order to achieve a smooth telemedicine experience. Promoting patient engagement through effective communications, improving patient-physician interactions, and paying more attention to the positive clinical outcomes, such as decreased anxiety and pain, are necessary to encourage patients and their healthcare providers to use telemedicine services more effectively, despite ongoing challenges posed by the pandemic.

The experiences obtained during the Covid-19 pandemic can also contribute to plan for the future and better use of the telemedicine technology. In fact, by leveraging the recent literature, evidence-based findings, and best practices in telemedicine implementation, this technology can be adopted more effectively and broadly in cancer care in the post-Covid era, as its weaknesses have been previously identified and now, they need to be addressed properly. Furthermore, as patients and healthcare providers are relatively ready to accept the technology, team working and establishing interdisciplinary collaborations among oncologists, information technology experts, and policymakers can help to develop new implementation strategies tailored to the specific needs of cancer patients to promote the adoption of telemedicine technology.

### Strengths and limitations of the study

This scoping review enhanced our understanding about using telemedicine for cancer care during the Covid-19 pandemic through a thorough examination of different technologies, their applications, and outcomes. Presenting users’ perspectives and experiences with telemedicine technology, challenges and clinical outcomes compared with the results of the in-person visits were other strengths of this study which can make a substantial contribution to the ongoing endeavors aimed at enhancing and promoting the consistent utilization of telemedicine services in cancer treatment.

However, this study had some limitations, including the restriction of articles to the English language. In fact, due to time and resource constraints, publications in other languages were not included. Moreover, this research solely focused on the interventional studies that analyzed the outcome of telemedicine technology and compared the results with the in-person visits. Therefore, other types of the research studies were excluded from the current study. Future studies can broaden their scope by incorporating various types of studies and objectives into their design to gain an in-depth understanding of the use of telemedicine for different groups of patients and healthcare providers.

In contrast to systematic reviews, the scoping review did not formally evaluate the methodological quality or risk of bias of the included studies. This constraint directly results from the fundamental nature of scoping reviews, which primarily focus on mapping the existing evidence rather than critically evaluating individual studies. Nevertheless, future studies could incorporate quality assessment methods to enhance the credibility of the results by conducting systematic reviews.

## Conclusion

This study aimed to investigate the use of telemedicine technology for cancer patients during the Covid-19 pandemic. The research findings provided a valuable insight into the use of various telemedicine technologies, services, and users’ opinions about, and clinical outcomes of these services. The results showed that in most studies positive outcomes in terms of patients’ and providers’ satisfaction and clinical aspects were reported after using telemedicine interventions. This shows that overall, the context is ready to accept the technology and there are a number of opportunities for expanding the use of telemedicine technology, particularly for cancer patients. Therefore, future research should focus on identifying optimal strategies for implementing telemedicine for cancer care while taking into account both the clinical and non-clinical effectiveness of various services and technologies.

## Data Availability

All data generated or analyzed during this study are included in this published article.

## Abbreviations

IARC: International Agency for Research on Cancer
PRISMA ScR: Preferred Reporting Items for Systematic reviews and Meta-Analyses extension for Scoping Reviews
